# Isolated Hypoglossal Nerve Palsy Secondary to Oligosecretory Multiple Myeloma

**DOI:** 10.7759/cureus.33361

**Published:** 2023-01-04

**Authors:** Bahaar K Muhar, Shawn Cho, Ashwin S Sidhu, Jason Chang, Forshing Lui

**Affiliations:** 1 Neurology, California Northstate University College of Medicine, Elk Grove, USA; 2 Neurology, Kaiser Permanente South Sacramento Medical Center, Sacramento, USA; 3 Clinical Sciences, California Northstate University College of Medicine, Elk Grove, USA

**Keywords:** oligosecretory, free light chains, hypoglossal nerve, lytic bone lesion, multiple myeloma

## Abstract

Isolated hypoglossal nerve (CN XII) palsy is rare. Neurological complications of multiple myeloma (MM) are quite common, most often due to hyperviscosity and paraprotein-related neuropathy. Direct compression of CN XII can be caused by plasmacytoma, yet direct invasion by MM is extremely rare. We are reporting a very unusual case of a 45-year-old man who presented with an isolated right CN XII palsy. The cause revealed by MRI is stenosis of the hypoglossal canal resulting from lytic bony erosion. Despite negative serum and urine protein electrophoresis tests, the final diagnosis of oligosecretory MM was confirmed by serum-free light chain test and bone marrow biopsy. The causes and diagnosis of isolated XII nerve palsy and oligosecretory MM are discussed.

## Introduction

Hypoglossal nerve (CN XII) lesions result in tongue muscle paralysis. Three different parts of the hypoglossal nerve pathway are susceptible to injury: the CN XII itself, the supranuclear region (above CN XII nuclei), and the motor axons [1]. Isolated hypoglossal nerve palsy is characterized by ipsilateral tongue deviation caused by tongue muscle weakness [1]. Due to the close proximity of CN XII to other cranial nerves and extensive anatomical path, isolated CN XII palsy is uncommon. Bilateral involvement is frequently related to nerve degeneration or demyelination, whereas unilateral involvement usually indicates compressive lesions [1].

Around 10% of hematologic malignancies are multiple myelomas (MM), often presenting with complications associated with renal failure, anemia, hypercalcemia, and bone pain [2]. Neurological findings are less commonly associated with MM, but when they are, it is usually as peripheral neuropathy or results of hyperviscosity [2]. Compression of CN XII has previously been shown due to plasmacytomas but not MM [3]. In this report, we describe an unusual case of a 45-year-old man who presented with an isolated CN XII palsy caused by oligosecretory MM with negative serum and urine protein electrophoresis (SPEP and UPEP).

## Case presentation

A 45-year-old male was referred to our neurology clinic because of two weeks history of difficulty swallowing and slurring of speech. He had no significant past medical history except recurrent brief episodes of low back pain. He presented to his primary care doctor six weeks prior due to worsening and persistent low back pain. The pain was worse with movement and weight lifting. He denied any trauma, extremity weakness, or loss of bowel or bladder control. He has been using a walker for the past six weeks due to his back pain. He has no history of substance abuse. He smoked in the past and quit for ten years. His appetite has been good, with no weight loss. He also denied any recent sick contact or febrile illness. There is no history of recent travel.

A physical exam revealed tenderness over his lumbar spinous processes and paraspinal muscles. His spinal movement was limited due to pain. A neurological exam revealed that he was alert and well-oriented. His language function was normal. He had mild lingual dysarthria. He had a rightward deviation of his tongue without any fasciculations. Other cranial nerves and extremity neurological exams were otherwise normal.

The clinical diagnosis was right hypoglossal nerve palsy. A review of his lab results was significant for hypercalcemia, elevated BUN and creatinine, anemia, and a negative serum protein electrophoresis (SPEP) and urine protein electrophoresis (UPEP) (Table 1).

**Table 1:** Important Lab Results Lab results revealed mild anemia, hypercalcemia, and elevated BUN and creatinine.

Test	Value	Normal
Hemoglobin	10.4 (low)	12 – 18 g/dL
White Blood Cell Count	5,600	4,000 – 11,000/mL
Platelet Count	206,000	150,000 – 400,000/mL
Blood urea nitrogen	32 (high)	8 – 21 mg/dL
Creatinine	1.42 (high)	0.6 – 1.2 mg/dL
Calcium	11.8 (high)	8.5 – 10.7 mg/dL
Serum Protein Electrophoresis	normal	
Urine Protein Electrophoresis	normal	

X-ray of the spine taken prior to the neurology consult revealed severe osteopenia with multiple thoracic and lumbar vertebral body compression fractures. Given these findings, an underlying malignancy, especially hematological malignancy, was suspected, and MM was seriously considered yet deemed unlikely by the hematologist, given his negative SPEP and UPEP findings. Right CN XII nerve malignant infiltration was considered a likely cause for his hypoglossal palsy.

**Figure 1:**
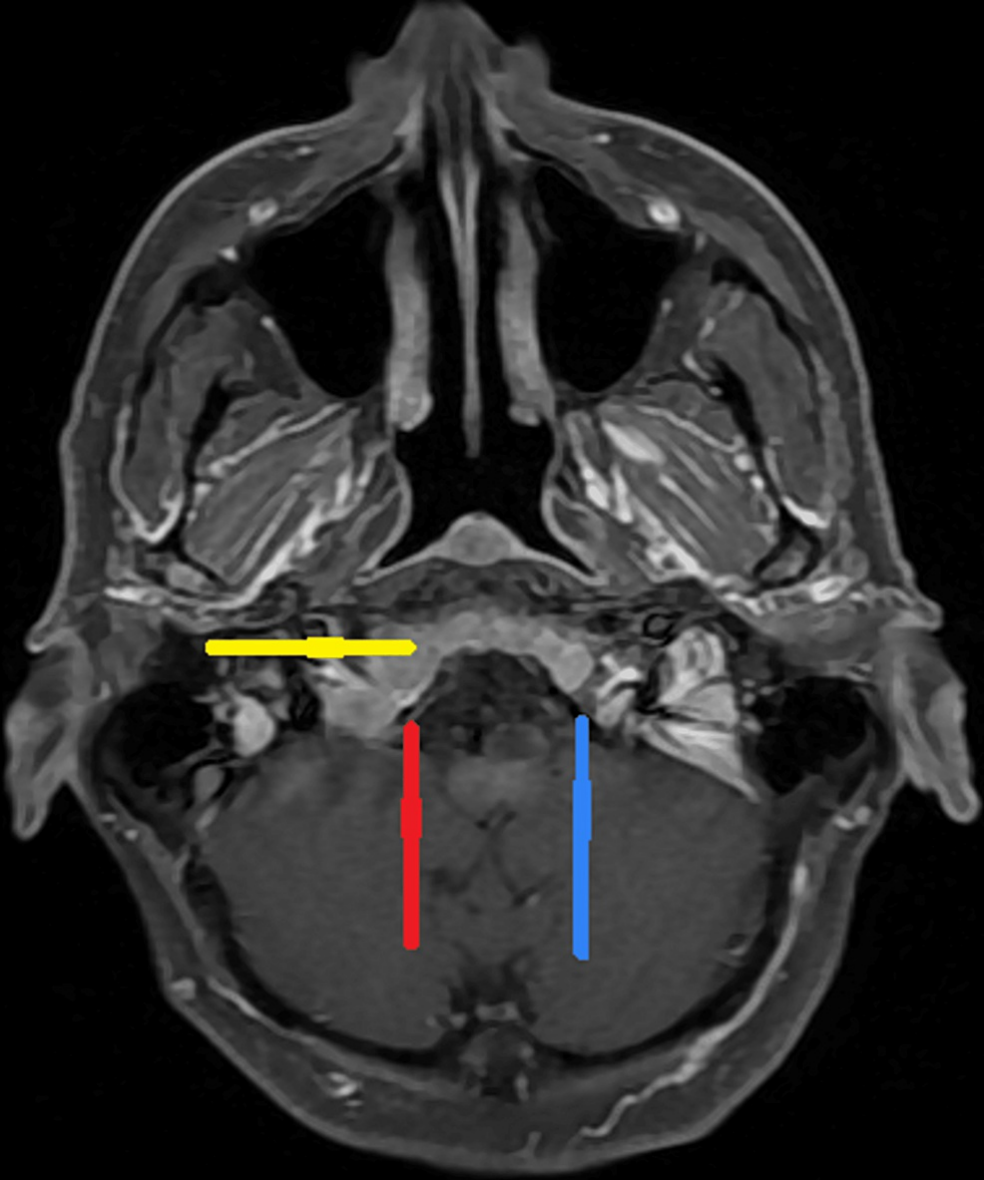
MRI revealing right hypoglossal canal narrowing due to diffuse bone marrow expansion. Axial MRI showing stenotic right hypoglossal canal (red arrow), the normal left hypoglossal canal (blue arrow), and diffusely abnormal bone marrow signal in clivus (yellow arrow)

Subsequent MRI of the head revealed diffuse, abnormal marrow with expansion in the skull base and visible upper cervical spine with narrowing of the right hypoglossal canal, causing compression and paresis of the right XII nerve (Figure 1).

A bone marrow biopsy obtained later confirmed histologically MM. Serum free light chains (FLC) then showed a kappa light chain value of 548 mg/L, a lambda light chain value of 6 mg/L, and a ratio of 83, confirming the diagnosis of oligosecretory kappa light chain MM. Subsequently, the patient’s management was taken over by his oncologist.

## Discussion

Due to the cranial nerve CN XII’s close anatomical relationship to other medullary pathways and nuclei with the other lower CN in their path outside the medulla oblongata, CN XII palsy usually presents with simultaneous involvement of other CNs such as CN IX and X or spinal accessory nerves (CN XI). CN XII may be affected at any point in its length; therefore, precise knowledge of the path and its relationship with other structures is necessary [4]. Isolated CN XII palsy is rare, and about half of the cases are idiopathic [5]. A prior study indicated cancer, trauma, and ischemia were most commonly associated with isolated CN XII palsy [6]. The most helpful test would be imaging along the entire course of the nerve. The MRI sheds light on the cause of our patient’s XII nerve palsy. It was the result of bony impingement and was the initial indication of an advanced bone marrow infiltration caused by MM, a very unusual cause.

In addition to symptoms of bone pain, weight loss, fatigue, lab findings of anemia, and X-ray abnormalities, a diagnosis of MM is usually made based on the presence of monoclonal protein on serum or urine immunoelectrophoresis (SPEP and UPEP). Oligosecretory MM caused by the monoclonal light chain, as in our patient, is rare [7]. In our patient, the SPEP and UPEP were normal. This is the time when testing specifically for serum and urine-free light chains is important in helping to make the diagnosis [8]. Intracranial manifestation of MM is uncommon. The most frequent intracranial manifestation of MM is dural involvement, which is caused by the direct extension of a plasmacytoma of the skull into the CNS [9]. It is even more unusual to have bony erosion or replacement due to his MM causing stenosis of the hypoglossal canal and the XII nerve palsy. We could find only a single case report in the literature describing an isolated XII nerve palsy caused by lytic bony lesions in MM [6]. The combination of his clinical picture of bone pain, anemia, hypercalcemia, and the bony changes in MRI causes our high index of suspicion of an underlying MM despite his negative SPEP and UPEP. The serum light chain essentially confirmed the diagnosis, which is finally confirmed by bone marrow tissue biopsy.

## Conclusions

Isolated hypoglossal nerve palsy is rare. Imaging the whole course of the nerve is the best diagnostic test. MRI of the head with visualization of the nerve along its course leads us to the cause in our patient (i.e., severe narrowing of the hypoglossal bony canal). Isolated hypoglossal nerve palsy caused by MM secondary to lytic bony changes has been described only once in our search of the literature. The usual diagnostic tests for MM or monoclonal gammopathy are SPEP and UPEP. Our case demonstrated the importance of serum and urine-free light chain tests when UPEP and SPEP are negative to help in the diagnosis of oligosecretory MM. Our case also additionally emphasizes the importance of looking at the whole clinical picture in order to arrive at a correct diagnosis.
